# Triangulating nutrigenomics, metabolomics and microbiomics toward personalized nutrition and healthy living

**DOI:** 10.1186/s40246-023-00561-w

**Published:** 2023-12-08

**Authors:** George Lagoumintzis, George P. Patrinos

**Affiliations:** 1 Division of Pharmacology and Biosciences, Department of Pharmacy, School of Health Sciences, University of Patras, 26504 Patras, Greece; 2 Department of Genetics and Genomics, College of Medicine and Health Sciences, United Arab Emirates University, Al-Ain, Abu Dhabi, UAE; 3 Zayed Center for Health Sciences, United Arab Emirates University, Al-Ain, Abu Dhabi, UAE

**Keywords:** Genomics, Genotyping, Healthy living, Microbiomics, Metabolomics, Nutrigenomics, Personalized nutrition

## Abstract

The unique physiological and genetic characteristics of individuals influence their reactions to different dietary constituents and nutrients. This notion is the foundation of personalized nutrition. The field of nutrigenetics has witnessed significant progress in understanding the impact of genetic variants on macronutrient and micronutrient levels and the individual's responsiveness to dietary intake. These variants hold significant value in facilitating the development of personalized nutritional interventions, thereby enabling the effective translation from conventional dietary guidelines to genome-guided nutrition. Nevertheless, certain obstacles could impede the extensive implementation of individualized nutrition, which is still in its infancy, such as the polygenic nature of nutrition-related pathologies. Consequently, many disorders are susceptible to the collective influence of multiple genes and environmental interplay, wherein each gene exerts a moderate to modest effect. Furthermore, it is widely accepted that diseases emerge because of the intricate interplay between genetic predisposition and external environmental influences. In the context of this specific paradigm, the utilization of advanced "omic" technologies, including epigenomics, transcriptomics, proteomics, metabolomics, and microbiome analysis, in conjunction with comprehensive phenotyping, has the potential to unveil hitherto undisclosed hereditary elements and interactions between genes and the environment. This review aims to provide up-to-date information regarding the fundamentals of personalized nutrition, specifically emphasizing the complex triangulation interplay among microbiota, dietary metabolites, and genes. Furthermore, it highlights the intestinal microbiota's unique makeup, its influence on nutrigenomics, and the tailoring of dietary suggestions. Finally, this article provides an overview of genotyping versus microbiomics, focusing on investigating the potential applications of this knowledge in the context of tailored dietary plans that aim to improve human well-being and overall health.

## Introduction

Nutrition refers to the biochemical and physiological processes through which an organism obtains sustenance from food. It facilitates the delivery of digestible nutrients to organisms, thereby supplying them with energy and chemical structures essential for their survival, growth, and reproductive processes [1]. Cells use nutrients in metabolic biochemical reactions to transform precursor metabolites into building block molecules. These molecules are then assembled into macromolecule polymers and subsequently utilized to construct complex and functional cellular structures. These structures, including carbohydrates, proteins, and fats, play crucial roles in the organism's overall life, well-being, and longevity.

The advent of the chemical revolution in the late nineteenth century marked a significant turning point in the scientific analysis of food and nutrition. The commencement of contemporary nutrition science was initiated in the 1910s with the identification of numerous micronutrients [2]. Thiamine, known as vitamin B1, was the first to undergo chemical isolation in 1926. Subsequently, in 1932, the preventive properties of vitamin C against scurvy were discovered [3, 4]. In the ensuing decades, researchers in the field of nutrition have conducted additional investigations and provided further clarification on the importance of vitamins and other essential nutrients [5]. The field of medicine and healthcare has undergone significant transformations since the beginning of the twentieth century, primarily driven by advancements in technology, biomedical and clinical research, and improved capacity for disease management [6, 7]. Over time, there has been the emergence of novel paradigms in the field of medicine and healthcare. These paradigms aim to prioritize individual-centric approaches, which are technically feasible and hold economic value while being ethically and socially accepted. In an era characterized by a growing desire for consumer customization, it is increasingly evident that advancements in the realms of research, management, and implementation of personalized medicine, therapy, and nutrition hold the potential to enhance both the longevity and quality of human life significantly. Moreover, such progress aims to optimize resource utilization and empower individuals with the autonomy to actively participate in their healthcare decision-making processes.

Personalized nutrition refers to the practice of tailoring dietary advice and interventions to accommodate an individual's distinct nutritional requirements, genetic composition, health condition, lifestyle, and personal preferences [8]. The meaning of the term acknowledges the individual variability of nutritional needs, which is influenced by a range of factors (such as age, gender, body composition, metabolic rate, genetic variations, and health considerations), and endeavors to offer customized and efficacious dietary recommendations aimed at enhancing health outcomes [9]. In general, personalized nutrition can revolutionize our approach to diet and nutrition by customizing nutritional guidance based on individual characteristics. The significance of personalized nutrition is anticipated to increase to achieve optimal health and well-being, given the advancements in our comprehension of genetics, metabolism, and nutrition [10].

Currently, state-of-the-art molecular biology technologies and traditional methodologies assist us in rapidly uncovering the significance of novel scientific concepts and theories in the progression of our scientific understanding. This assertion holds particular importance in the context of the microbiome and genotyping concept [11, 12]. The gut microbiome has garnered significant attention in the scientific community over the past decade due to its intricate and indispensable role in human health [13]. The interactions between the microbiome and the host exhibit high complexity and involve multiple facets. These interactions significantly shape the host's overall health and well-being. There is an interaction between the intestinal microbiota and the immune system, along with other major body systems and organs, such as the gut-brain axis and gut-(other organs) axis [14]. Consequently, this can affect the equilibrium between health and disease.

While certain factors originating from the host are innate and challenging to alter, the microbiome can be more easily influenced by environmental factors, particularly dietary choices [15, 16]. It is becoming increasingly acknowledged that the microbiome can impact human physiology through its involvement in digestion, nutrient absorption, the development of the mucosal immune system, and the production or adjustment of numerous potentially biologically active substances. Therefore, alterations in the microbiota caused by dietary factors can be utilized to provoke physiological modifications in the host, which may include the onset and advancement of diseases. Various interventions have been employed to manipulate the composition and activity of the microbiome, including the administration of probiotics, utilization of prebiotics, implementation of dietary modifications, application of fecal microbiota transplantation (FMT), and the utilization of targeted antimicrobial therapies [17–19]. Nevertheless, there are notable constraints in the processing and analyzing of large datasets, which hinder our ability to interpret and translate the obscure connections between hosts, microbiomes, and diets at an individual level. Undoubtedly, the microbiome is a complex and individualized entity, necessitating further investigation to comprehend its intricacies and potential for tailored health interventions systematically. However, the field of microbiome research is rapidly expanding, bolstered by advanced technologies like DNA next-generation sequencing (NGS), which enables comprehensive analysis of microbial communities.

The primary aims of this article are to provide an overview of the most critical developments and insights pertaining to the principles of personalized nutrition, with a specific focus on the intricate interplay between genes, diet metabolites, and the microbiota, the distinctiveness of gut microbiota composition, and its impact on nutrigenomics and individualization of diets. Additionally, this article outlines the field of genotyping toward exploring the potential applications of this knowledge in the context of personalized nutrition strategies to enhance human health and overall well-being.

### Personalized nutrition

#### Concept of nutritional personalization

**Fig. 1:**
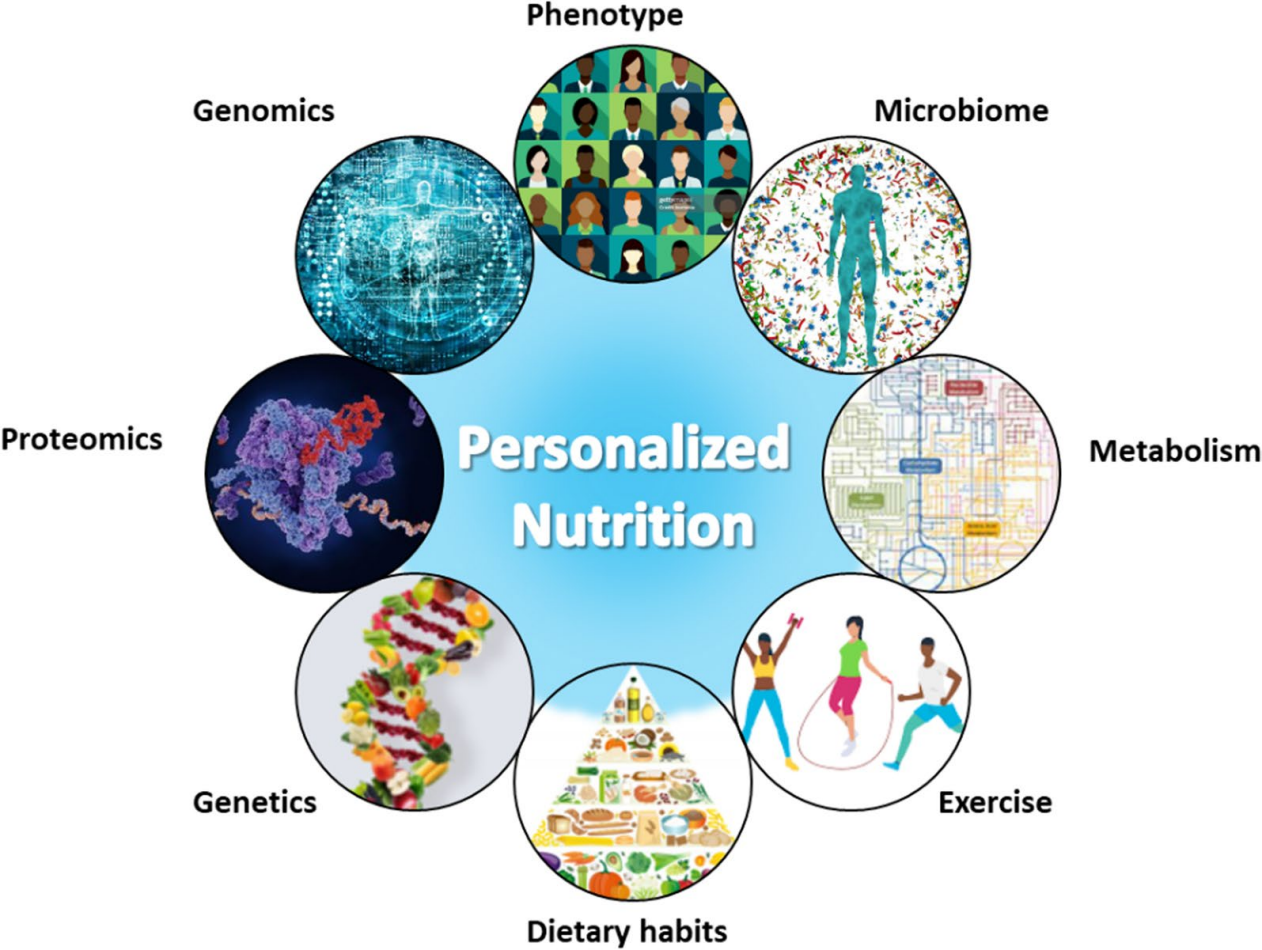
Aspects of personalized nutrition concept affecting individual’s healthy life and well-being

When making decisions regarding the optimal treatment plan for a patient, personalized medicine considers the individual's distinct genetic composition and other pertinent biological attributes [20]. In recent years, technological advances have been made, specifically in genetic testing and genomic analysis. These advancements have facilitated the identification of specific gene variants that have the potential to influence drug metabolism, responsiveness to medical treatments, and susceptibility to certain diseases. These technologies offer crucial data that can aid healthcare professionals in making more informed decisions regarding the medications they prescribe, their dosages, and their treatment modalities. Similarly, the concept of personalized nutrition recognizes that individuals possess distinct physiological and genetic characteristics that impact their responses to different types of foods and nutrients (Fig. 1) [8].

Personalized nutrition has the potential to aid individuals in attaining and sustaining a healthy body weight, optimizing their nutritional intake, effectively managing chronic illnesses, enhancing sports performance, and promoting overall well-being. The utilization of genetic testing and biomarker analysis can provide valuable insights into an individual's genetic variations pertaining to nutrient metabolism, food intolerances and sensitivities, and other facets of nutrition. By incorporating this data alongside other factors such as age, gender, body composition, and pre-existing health conditions, it becomes feasible to develop personalized dietary interventions aimed at optimizing overall health results [21–24]. Both personalized medicine and personalized nutrition share a common overarching goal, which is to tailor medical procedures according to an individual's distinct characteristics. Personalized nutrition involves tailoring dietary recommendations to align with an individual's specific nutritional requirements, genetic composition, state of health, lifestyle, and personal preferences. This stands in opposition to the objective of personalized medicine, which aims to enhance medical interventions by taking into account solely an individual's genetic makeup.

According to the findings of Micha et al. [25], it is widely acknowledged that dietary factors play a significant role in the development of various prevalent diseases, including but not limited to cardiovascular diseases (CVDs), type 2 diabetes mellitus (T2DM), and cancer. Moreover, to optimize public health outcomes, it is feasible to integrate a simultaneous application of dietary management within the context of personalized nutrition for populations with distinct nutritional requirements, such as lactating mothers, pregnant women, elderly individuals, and individuals in a state of well-being. Indeed, according to Pickering and Kiely [26], individuals who possess certain personal objectives, such as attaining a specific physique or size, engaging in competitive athletic activities, or managing dietary preferences, also pursue personalized nutrition strategies.

As further indicated by Ordovas et al. [27], these findings suggest the potential for individualized dietary practices to exert a more substantial influence on behavior modification and ultimate health outcomes. The concept of "personalized nutrition" pertains to a strategic approach that entails the development of a tailored set of nutritional recommendations, products, or services, taking into account the unique characteristics of an individual. The term "personalized nutrition" is often referred to as "precision nutrition" in academic literature [28]. According to Gibney et al. [29], this approach facilitates individuals in attaining a durable modification in dietary behavior that is advantageous for their overall health. There is a certain degree of convergence between the notions of “personalized nutrition” and related terms such as “precision nutrition,” “nutrigenomics,” “personalized nutrition,” “tailored nutrition,” and “stratified nutrition.” Specific terms in this context exhibit interchangeability, as exemplified by the synonymous usage of "customized nutrition" and "personalized adapted nutrition." The primary objective of all terms is to offer suitable dietary recommendations to specific individuals [8, 30, 31].

#### The human microbiome as a focal point for personalized medicine and nutrition

The term "microbiome" pertains to the collective genetic material of all bacteria present in a specific ecological setting, while "microbiota" denotes the assemblage of microorganisms within that community. The human microbiome is the collection of microorganisms that inhabit and colonize the body's various surfaces and internal regions. The category of microorganisms encompasses various types such as bacteria, viruses, fungi, and other microbial entities [32]. The human gastrointestinal (GI) tract harbors a vast number of bacteria (over 5–10 trillion), constituting a highly sophisticated ecosystem that plays a pivotal role in various essential biological processes [33]. These include the digestion and absorption of nutrients, regulation of the immune system, and exerting influence on mental well-being and cognitive function [12, 34]. These microorganisms collectively constitute the host's microbiome and are now acknowledged as a distinct organ within the human body. The microbiome exerts a significant influence on overall health and well-being by possessing over three million genes. These genes produce numerous metabolites that coordinate various functions within the host organism. Consequently, the microbiome profoundly impacts the host's fitness, phenotype, and overall health [35, 36].

The human microbiome has gained significant attention in personalized medicine due to its impact on disease development, treatment efficacy, and overall health results. Extensive research has established significant associations between the microbiome and a range of health conditions, encompassing obesity, diabetes, inflammatory bowel disease (IBD), allergies, mental health disorders, and many others [37–40]. Researchers are currently engaged in studying the microbiome to gain insights into the mechanisms through which bacteria impact human health. Within the framework of personalized medicine, the microbiome has demonstrated its potential as a valuable biomarker for disease diagnosis, prognosis, and assessment of therapeutic response [41, 42]. Examining microbial composition and activity can enable healthcare providers to identify specific microbial signatures associated with various diseases or treatment outcomes coupled with food and nutrition. The data above can be employed to develop personalized interventions, including specific probiotics, prebiotics, or dietary modifications, to facilitate the restoration of a balanced microbiome and improve the overall outcomes of patients.

Moreover, the microbiota can potentially impact the metabolism and effectiveness of drugs [43, 44]. Certain microorganisms residing in the GI tract can modulate drug metabolism, potentially diminishing their efficacy or eliciting adverse reactions. Comprehending these interactions can facilitate choosing and administering medications for specific patients, thereby optimizing therapeutic benefits and minimizing adverse effects. Despite the potential of the microbiome in the field of personalized medicine, there are still substantial obstacles that need to be addressed. The development of standardized protocols for microbiome analysis and interpretation is underway, and extensive research is necessary to establish robust associations between microbial profiles and health outcomes. In addition, it is imperative to thoroughly contemplate ethical concerns, privacy concerns, and regulatory frameworks about the collection and analysis of microbiome data [45].

In summary, the human microbiome represents an up-and-coming area of focus within the field of personalized medicine that has the potential to revolutionize healthcare and improve patient outcomes. Personalized nutrition aims to tailor dietary recommendations according to an individual's specific microbiome composition and functionality, considering microbial diversity, metabolic capacity, and potential imbalances. The interaction between the microbiota and nutrition is dynamic and interrelated. The dietary choices made by individuals have the potential to impact the composition and functioning of the microbiome. In contrast, reciprocally, the microbiome influences food metabolism and absorption [46]. Understanding intricate associations between the microbiome and human health enables healthcare providers to formulate tailored approaches for preventing, diagnosing, and treating illnesses.

#### Microbiome’s fluctuations: causes and implications

Despite experiencing daily fluctuations, the composition of the gut microbiota is distinct to each individual and tends to exhibit relative stability throughout one's lifetime [47, 48]. The dietary factor is a variable component that has an impact on the makeup of the gut microbiota, suggesting the potential for therapeutic dietary interventions to modify the diversity, composition, and stability of microbial populations [49]. Markedly, the alteration of the gut microbiota can be influenced by dietary factors; however, it is important to note that these modifications seem transient. The question of whether sustained modifications in diet can result in persistent modifications in the gut microbiota remains uncertain, primarily due to the absence of extended human dietary interventions or prolonged monitoring of short-term dietary interventions. [50].

**Fig. 2:**
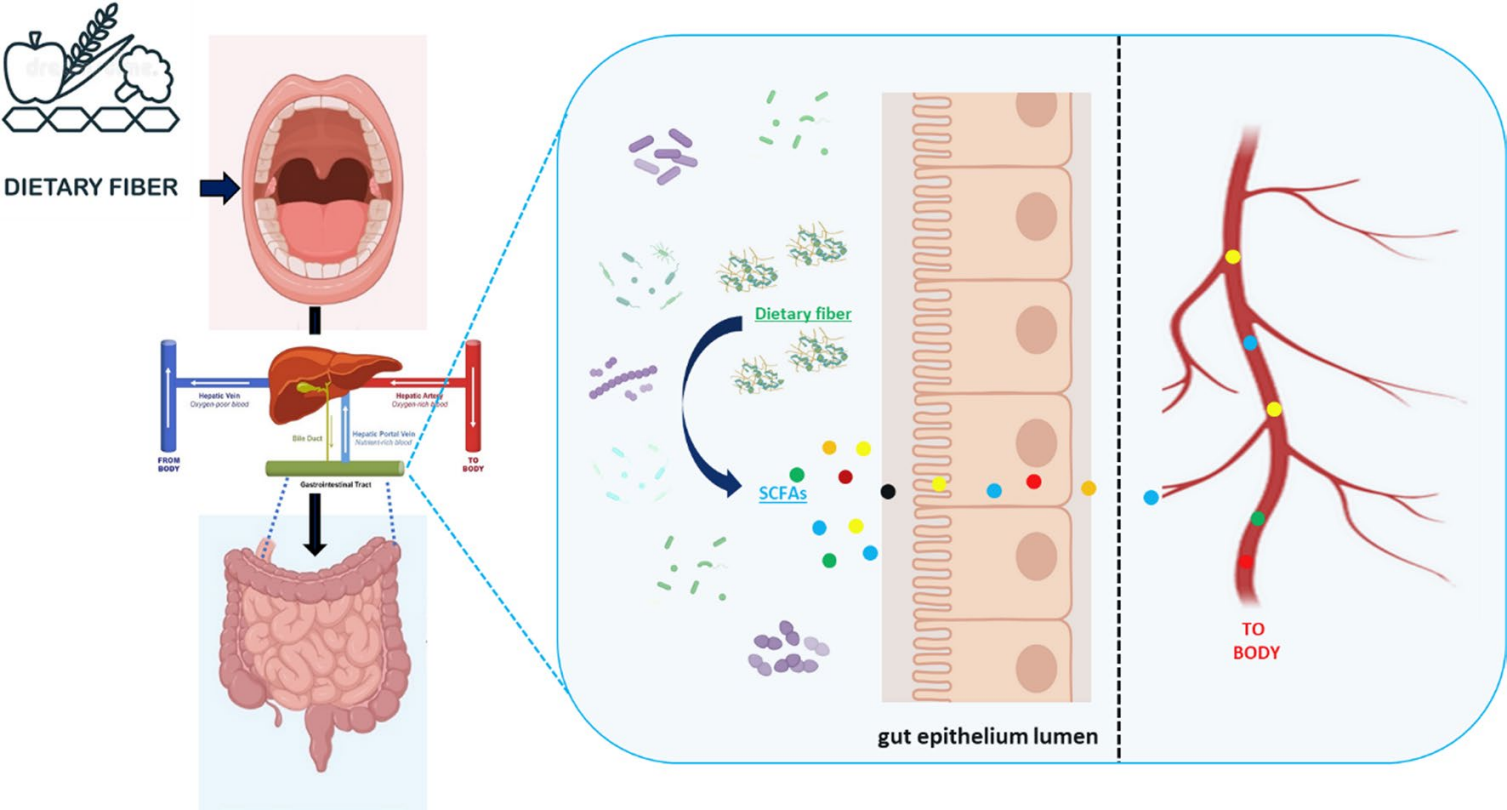
Dietary fiber intake and SCFA production

Dietary fiber, commonly present in fruits, vegetables, whole grains, and legumes, holds significant importance within a well-balanced dietary regimen and exerts a considerable influence on the microbiome [51, 52]. Fiber exhibits resistance to hydrolysis by endogenous human enzymes, thereby serving as a substrate for specific commensal bacteria residing in the GI tract. The production of Short-Chain Fatty Acids (SCFAs) is a consequence of fiber fermentation by these bacteria (Fig. 2). SCFAs provide cellular energy to the epithelium that lines the colon and offer a range of advantageous effects on health, such as mitigating inflammation, promoting GI well-being, regulating metabolism, and even brain communication [53]. Ingesting specific foods or supplements containing probiotics (*i.e.*, living beneficial bacteria) can potentially modify the microbiome. They possess the capacity to facilitate the maintenance of a favorable microbial equilibrium by aiding in the restoration or augmentation of particular beneficial bacteria within the GI tract.

While the field of personalized nutrition and its impact on the microbiome is still in its promising phase, several key principles can be adhered to promote a healthy microbiome through dietary interventions. Incorporating a diverse range of fruits, vegetables, whole grains, and legumes into one's diet to obtain a wide array of fiber sources, reducing the consumption of processed meals and added sugars, and including fermented foods like yogurt, kefir, sauerkraut, and kimchi, which harbor live beneficial bacteria, exemplify these practices. In contrast, a suboptimal dietary pattern characterized by insufficient fiber intake, excessive consumption of processed foods, added sugars, and unhealthy fats may harm the composition and function of the microbiota [54–58]. Accordingly, this trend, among other human habitual and cultural preferences, has the potential to result in a decrease in the variety of microbial species and an alteration in the makeup of the microbial community, a condition commonly known as dysbiosis. Dysbiosis has been associated with a range of health conditions, encompassing inflammation, obesity, metabolic irregularities, and GI disorders [59, 60].

In addition, it should be noted that the microbiome can modify the nutritional absorption and metabolism processes. Several bacterial species residing in the GI tract contribute to fat digestion through their ability to break down complex carbohydrates, synthesize vitamins, and metabolize bile acids. The microbial processes can potentially influence the accessibility and assimilation of nutrients within the food. Significantly, although nutrition can influence the microbiome, it should be noted that the microbiome is a multifaceted and diverse ecosystem subject to the influence of factors beyond dietary considerations. To this end, various factors, such as genetics, lifestyle choices, pharmaceutical utilization, stress levels, and environmental exposures, influence the composition and functioning of the microbiome [61]. Consequently, cultivating a comprehensive strategy toward health is imperative to foster an optimal microbiome, encompassing a well-balanced dietary regimen and a generally healthy way of life.

### The relationship between microbiomics and nutrient intake

#### Macronutrient intake

Dietary constituents are crucial in supplying our bodies with vital nutrients and acting as substrates for the symbiotic microflora residing in our GI tract. Various metabolites are produced through the conversion of undigested dietary components. Certain bacterial species present in the GI tract possess the capability to metabolize complex carbohydrates that are beyond the digestive capacity of our endogenous enzymes. These bacteria can synthesize cellulases and hemicellulases, enzymes that facilitate the conversion of fibrous macronutrients into SCFAs [62]. The gut microbiota is also implicated in the synthesis of various vitamins, such as biotin, folate, vitamin K, and specific B vitamins [63]. These vitamins are necessary for various physiological processes, including energy metabolism, DNA synthesis, and blood coagulation. Specific types of gut microbiota can undergo metabolic processes involving certain amino acids, producing advantageous byproducts such as SCFAs, ammonia, and indole compounds. These metabolites can regulate immune responses and impact brain function, as well as other effects on the physiology of the host [64].

The intestinal microbiota also influences the absorption of essential minerals such as calcium, iron, and magnesium. Certain bacterial species possess the ability to synthesize enzymes that aid in the degradation of mineral structures, thereby enhancing their bioavailability [65]. Furthermore, the composition and diversity of the GI microbiota can influence our food preferences, cravings, and appetite regulation [66, 67]. Several bacterial species can synthesize compounds that influence the synthesis of hormones involved in regulating appetite, such as leptin and ghrelin. This implies that alterations in the composition of the intestinal microbiota have the potential to impact the absorption and metabolism of nutrients, thereby playing a role in the development of conditions such as obesity and metabolic disorders [68].

The investigation of the correlation between GI microbiota and nutrient absorption is currently a vibrant field of research. Researchers are now examining the effects of specific dietary patterns, such as those high in fiber and supplemented with probiotics, on the modulation of the intestinal microbiota and the optimization of nutrient absorption [69]. To this end, utilizing personalized methodologies, such as microbiome sequencing and analysis, could potentially aid in identifying individuals possessing distinct microbiome profiles that may derive advantages from dietary interventions.

Carbohydrates, proteins, and fats are the primary macronutrients that serve as energy sources in human nutrition. However, it is important to note that their digestibility and nutrient profiles accessible to the microbiota exhibit considerable variation. The proliferation of specific bacteria and the production of specific metabolites in the gut epithelium and mucosal immune system are influenced by the quantity and composition of macronutrients, leading to either beneficial or detrimental effects [70]. Indigestible carbohydrates constitute a substantial portion of dietary fiber, and specific bacteria that can break down dietary fiber generate SCFAs. Dietary fiber can be classified into distinct categories, namely resistant starches, nondigestible oligosaccharides, nondigestible polysaccharides, and chemically synthesized carbohydrates, based on their chemical structures [71].

Dietary fiber is widely recognized as a crucial nutrient for maintaining a diverse GI microbiome [72]. A correlation has been observed between reduced microbial diversity and a range of chronic inflammatory conditions, such as obesity, diabetes, ulcerative colitis, and IBD [73–75]. Therefore, the ingestion of various fibrous substances has a significant impact on the structural, compositional, and functional aspects of the gut microbiome. This, in turn, interacts with the gut epithelium and mucosal immune system, playing a crucial role in maintaining a balanced and healthy intestinal environment.

#### Micronutrient intake

Apart from macronutrients, micronutrients play a crucial role in preserving the well-being of the host organism, encompassing both organic and inorganic substances, such as vitamins and minerals. Their deficiencies can result in notable health consequences, both in the short-term and long-term [76, 77]. Micronutrients are commonly found in both food sources and dietary supplements. Peptides play a crucial role in regulating biosynthetic cellular reactions, encompassing immune and energy functions and biological processes like growth, bone health, and fluid balance. Specific micronutrient deficiency is a significant global health concern, which can be attributed to the decreased intake and/or poor absorption of micronutrients in the GI tract, leading to reduced bioavailability. Micronutrient deficiencies can further contribute to the severity of infections and non-communicable chronic diseases, including osteoporosis, hypothyroidism, cardiovascular disease, and cancer [78, 79]. These deficiencies can significantly influence life expectancy, morbidity, and mortality outcomes. Emerging findings from clinical and in vivo studies indicate that the gut microbiome plays a significant role in the development of micronutrient deficiencies [80, 81].

The presence of commensal microorganisms can influence the production and absorption of micronutrients, thereby exerting control over their concentrations. In addition, microorganisms utilize micronutrients to support their growth and perform essential biological processes. Hence, it is unsurprising that the intake of micronutrients can impact the composition and functionality of the GI microbiota. Dietary supplementation with vitamins B, C, D, and E plays a significant role in shaping the composition of the microbiome by facilitating the expansion and colonization of beneficial genera such as *Bifidobacterium*, *Lactobacillus*, and *Roseburia* within the intestinal mucosa [82–85]. Minerals such as calcium, iron, zinc, magnesium, and phosphorus have the potential to exert an influence on the composition and functioning of the microbiome residing in the GI tract of humans [86, 87]. Specifically, elevated consumption of calcium is linked to an increased prevalence of *Clostridium* cluster XVIII in males [88]. Additionally, the administration of iron supplements may lead to a reduction in *Bifidobacterium* populations and an elevation in *Lactobacillus* levels among children [89]. Furthermore, the supplementation of phosphorous has been found to enhance the variety of microorganisms present and elevate the levels of SCFAs in fecal matter [88]. Conversely, previous studies have provided evidence that different constituents of the intestinal microbial community possess the ability to modulate the accessibility of micronutrients through the regulation of their absorption [90].

#### Modification of the gut microbiome

As previously discussed, the composition of the microbiome can be significantly influenced by various factors such as genetics, dietary patterns, lifestyle choices, substance consumption, exposure to environmental agents, and events occurring during early life stages (*i.e.*, breastfeeding). With the increasing recognition of the potential influence of the microbiome on human health, there has been a surge in interest in understanding and modulating its composition and functionality. Various interventions, such as probiotics, prebiotics, dietary modifications, FMT, and targeted antimicrobial therapies, are employed to manipulate the microbiome purposefully [91].

**Fig. 3:**
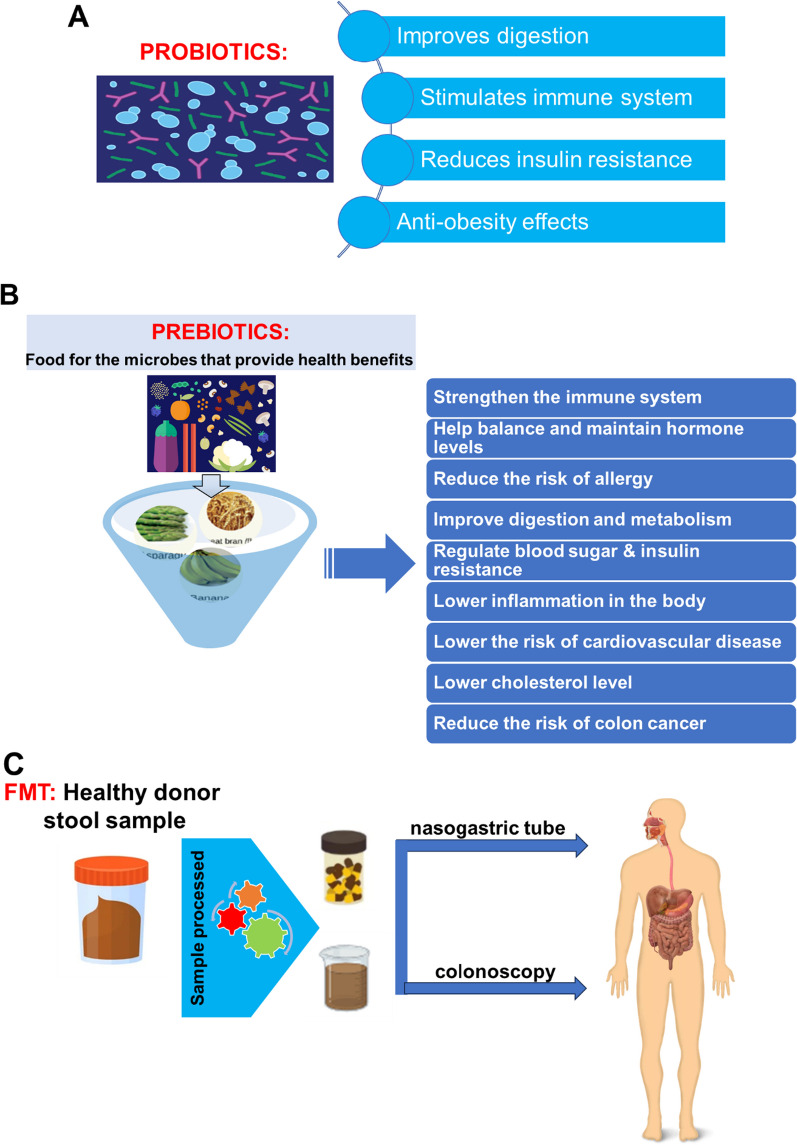
General aspects of the most common interventions in manipulating gut microbiome composition and its proper functionality. **A:** Probiotics, **B:** Prebiotics, **C:** Fecal Microbiota Transplantation (FMT)

Probiotics refer to microorganisms that are not pathogenic and do not cause diseases. When consumed in adequate amounts, these microorganisms stimulate advantageous effects in the host through multifactorial mechanisms (Fig. 3A) [92, 93]. In a clinical trial conducted on neonates, a group of infants was administered a mixture of pre/probiotics, while another group received a placebo. The results revealed that exclusively the neonates who received the pre/probiotic mixture exhibited weight gain [94]. On the other hand, the discovery of the lipid-lowering effects exhibited by various strains of *Lactobacillus* and *Bifidobacterium breve* in the context of diet-induced obesity suggests that probiotics have the potential to mitigate the accumulation of adipose tissue and decrease the body weight of the host [95–98]. As Delzenne and Reid assert the current body of evidence is inadequate to establish a definitive association between obesity and probiotics. Furthermore, they caution that findings from animal studies may not necessarily be applicable to human metabolism [99]. Hence, additional research is needed to explore the impact of probiotic supplementation on the energy regulation of the host, as the current body of evidence often presents conflicting findings.

Prebiotics refer to indigestible substances that, upon consumption, promote the proliferation of beneficial bacteria in the colon, including *Bifidobacteria* and *Lactobacilli* (Fig. 3B) [93, 100]. Oligofructose and inulin have been extensively studied as prebiotics in the context of weight regulation. Research findings have demonstrated that ingesting oligofructose leads to the production of peptide YY and glucagon-like peptide 1, which ultimately results in reduced food consumption and body weight in rodents. At the same time, in human subjects, they facilitate weight loss and promote feelings of fullness [101–105].

With respect to a more invasive and drastic method of altering the gut’s microbiota composition, the medical procedure known as FMT involves the transfer of feces from a healthy donor into the GI tract of a recipient, with the aim of restoring a healthy equilibrium of gut microbiota (Fig. 3C). The primary application of this treatment is in the management of dysbiosis, a condition characterized by an imbalance in the GI microbiome. It is particularly effective in addressing recurrent *Clostridium difficile* infection (CDI) [106]. Particularly, CDI is a bacterial infection that poses challenges in terms of treatment efficacy using conventional antibiotic therapies and is characterized by the manifestation of severe diarrhea. FMT has exhibited remarkable effectiveness in the management of recurrent CDI through the restoration of a healthy microbial community in the recipient's GI tract via the introduction of fecal material from a donor. However, ongoing investigations are being conducted to assess its effectiveness in addressing other conditions characterized by dysbiosis, such as IBD, irritable bowel syndrome, and other metabolic disorders [107–110]. Nevertheless, further investigation is necessary to ascertain the safety and effectiveness of FMT in diverse cohorts of patients.

### Microbiomics in nutrient-related pathologies

The gut microbiome plays a significant role in human health directly and indirectly through its interaction with diet [13, 111]. Differences in gut microbiome composition and function have been associated with various chronic diseases, including gastrointestinal inflammatory and metabolic conditions, neurological disorders, cardiovascular diseases, respiratory diseases, and cancer [39]. Diet greatly influences the microbiome of the digestive tract, as certain dietary components can promote or inhibit the growth of specific microbial species [112]. The gut's processing of macronutrients and micronutrients from the diet also regulates human responses to food types based on the microbiome's composition. Therefore, diet affects the host's health status by modulating the gut microbiome's composition and diversity [113, 114].

In terms of metabolic diseases, accumulating evidence suggests that the gut microbiome and its metabolites play a crucial role in the onset and development of conditions such as obesity, type 1 and type 2 diabetes, dyslipidemia, and nonalcoholic fatty liver disease (NAFLD) [115]. Studies have shown that individuals with obesity have different gut microbiome composition and function compared to lean individuals, with obese individuals being able to extract more energy from their diet [116, 117].

Liver diseases, such as NAFLD and alcoholic liver disease (ALD), are also influenced by the gut microbiome [118]. The gut and liver work in a bidirectional manner, and dysbiosis in the gut microbiota can contribute to developing liver disorders [119]. Clinical studies have shown a connection between gut microbiota dysbiosis and liver diseases, with changes in the composition of specific bacterial species [120–122]. However, further research is needed to determine whether dysbiosis is a cause or an effect of liver diseases.

CVDs are the leading cause of death globally, and the gut microbiome has been implicated in their development and pathophysiology. Trimethylamine-N-oxide (TMAO) production, a compound produced by gut microbes from specific dietary components, is associated with cardiovascular events [123]. Studies have found differences in the gut microbiome composition between individuals with CVD and those without, with lower levels of *Bacteroidetes* and higher levels of *Lactobacillales* in CVD patients [124]. Furthermore, recent studies have demonstrated that metabolites originating from the gut, specifically SCFAs, can influence blood pressure regulation [125].

The gut-brain axis plays a role in neurodegenerative disorders like Parkinson's disease (PD) and Alzheimer's disease (AD). Modifications in the gut microbiome have been observed in individuals with these disorders, with specific bacterial species potentially playing a role in their development. Studies have shown differences in the gut microbiome composition between individuals with PD and healthy controls, and FMT from healthy donors has shown transient improvements in PD symptoms [126]. Moreover, various mental disorders are distinguished by the distinct composition of gut microbiota, typically dominated by a specific bacterial genus or family. It is worth noting that certain disorders exhibit an excessive presence of particular microbial species. For instance, Zhu et al. [127, 128] have reported a notable presence of *Lactobacillus sp.* in individuals with schizophrenia.

The microbiome can also impact cancer development and treatment. While only a few microorganisms have been officially recognized as causes of cancer in humans, recent research suggests that numerous microbial species may influence or participate in cancer development [129–137]. Although the causal evidence of microbial influences on cancer biology is still being explored, there is a growing recognition of the significance of comprehending the molecular aspects of these interactions and their effects on cancer treatment. The comprehensive summary of research findings on the impact of the microbiome on cancer falls beyond the purview of this review. However, interested readers may find illumination in various existing reviews on the subject within the field of cancer [137–141].

Overall, the gut microbiome significantly impacts nutrient-related pathologies, including metabolic diseases, liver diseases, cardiovascular diseases, neurodegenerative disorders, and cancer. Yet, additional research is needed to fully understand these associations' mechanisms and develop targeted interventions for improving human health. Table 1 summarizes some of the most common nutrient-related human diseases affected by gut microbiome function and diversity with annotated indicative references for further reading.

**Table 1:** Overview of some of the most common nutrient-related human pathologies linked with gut microbiome abundance and functionality

Human nutrient-related pathologies	Gut species related/affected (↓: reduced, ↑: increased)	Indicative associated refs.
NAFLD	↑ *Escherichia coli* ↑ *Bacteroides vulgatus* ↑ *Bacteroides* ↑ *Ruminococcus*	[142–145]
	↓ *Prevotella*	
IBD (Crohn’s disease and ulcerative colitis)	↑ *Bacteroides fragilis* ↑ *Ruminococcus torques* ↑ *Ruminococcus* ↑ *Clostridium hathewayi* ↑ *Clostridium bolteae* ↑ *Ruminococcus gnavus* ↑ *Actinomyces* ↑ *Veillonella* ↑ *Intestinibacter*	[146–155]
	↓ *Bifidobacterium longum* ↓ *Eubacterium rectale* ↓ *Faecalibacterium prausnitzii* ↓ *Roseburia intestinalis* ↓ *Christensenellaceae* ↓*Coriobacteriaceae* ↓ *Clostridium leptum* ↓ *Eubacterium rectum* ↓ *Akkermansia muciniphila* ↓ *Coprococcus* ↓ *Blastocystis*	
CVDs	**↑** *Proteobacteria* **↑** *Actinobacteria* *Prevotella* ↑ *Erwinia* ↑ *Corynebacteriac-eae* ↑ *Firmicutes/Bacteroides ratios* ↑ *Streptococcus* ↑ *Enterobacteriaceae* ↑ *Lactobacillales* ↑ *Clostridium subcluster* XIVa ↑ *Campylobacter* ↑ *Candida* *↑ Shigella* ↑ *Salmonella* ↑ *Yersinia Enterocolitica* ↑ *Escherichia/Shigella* *↑ Klebsiella pneumonia* *↑ Streptococcus viridians*	[156–163]
	↓ *Anaerostipes* ↓ *Lactobacillus murinus* ↓ *Bacteroides* **↓** *Roseburiam*	
ALD (and cirrhosis)	↑ *Proteobacteria* ↑ *Lactobacillus*/*Bifidobacterium* ↑ *Streptococci* ↑ *Enterobacteria* ↑ *Bifidobacterial* ↑ *Akkermansia muciniphila*	[164–168]
	↓ *Ruminococcaceae* ↓ *Bacteroidetes*	
CKDs	**↑** *Enterobacteriae* ↑ *Enterococci* ↑ *Lachnospiraceae* ↑ *Ruminococcaceae* ↑ *Gammaproteobacteria* ↑ *Actinobacteria* ↑ *Firmicutes* ↑ *Clostridium* ↑ *Enterococcus* ↑ *Pseudomonas aeruginosa*	[169–178]
	↓ *Lactobacillaceae* ↑ *Prevotellaceae* ↓ *Bacteroidaceae* ↓ *Bifidobacterium* ↓ *Lactobacillaceae* ↓ *Prevotellaceae* ↓ *Actinobacteria* ↓ *Lactobacillaceae* ↓ *Bifidobacterium* ↓ *Actinobacteria*	

*NAFLD* Non-alcoholic fatty liver disease, *IBD* Inflammatory bowel disease, *CVDs* Cardiovascular diseases, *ALD* Alcoholic liver disease, *CKDs* Chronic kidney diseases

### Metabolomics and microbiomics in personalized nutrition

Metabolomics studies metabolites, which are biologically small molecules with a molecular weight of less than 1,500 Daltons. The analysis of these metabolites occurs within biological fluids, tissues, and cells over a predetermined period of time and in response to particular environmental conditions. Metabolomics is an essential constituent of the 'omics' fields, as it provides biochemical insights in conjunction with genomic and proteomic data [179, 180], thereby establishing a direct correlation with the phenotype of an organism. Nutritional metabolomics is an essential component of the metabolomics discipline as it evaluates the unique functional responses of individuals to different diets, investigates specific dietary biomarkers linked to particular foods and diets, and explores the interrelationships between diverse diets and risk factors for specific diseases in the veterinary and human sciences [181, 182]. The primary aim of nutritional metabolomics technology is to evaluate the distinct reactions of organisms, including humans and animals, to various dietary components. Its objective is to identify and implement individualized nutritional strategies that foster optimal health [181, 183].

Metabolomics generally classifies methodologies into two primary categories: untargeted and targeted techniques. A methodology known as targeted metabolomics identifies particular metabolites through the process of comparing them to predetermined standards. This methodology proves to be advantageous in the pursuit of biomarker development and hypothesis testing [184]. On the other hand, untargeted metabolomics is predominantly concerned with the identification of molecules that have not been previously known [185]. Targeted metabolomics has garnered considerable interest as the need to identify and quantify biologically active compounds has increased. Significantly implemented in the domain of diet and nutrition [184, 186], this methodology has a wide range of practical implications. For example, it is capable of identifying biomarkers of food intake [187] and detecting nutritional disorders or deficiencies [188]. Furthermore, targeted metabolomics is utilized in the examination of food composition, estimation of dietary intake [189], and provision of appropriate recommendations for the management of chronic diseases [190].

#### Dietary biomarkers and metabolomics

A comprehensive understanding of an individual's overall nutritional status and dietary consumption is an essential foundation in the field of precision nutrition. In the past, the assessment of an individual's nutritional health encompassed a variety of methodologies, including surveys, dietary diaries, 24-h dietary recalls, and food frequency questionnaires. However, a number of limitations are applicable to these approaches. A variety of obstacles are present in the realm of food consumption reporting, including deliberate misrepresentation, recall bias, memory constraints, and difficulties in precisely calculating portion sizes. The aforementioned limitations may lead to the accumulation of erroneous or inconsistent data, thereby introducing unpredictability into the search for dietary biomarkers. As a result, the utilization of analytical instruments to precisely evaluate a person's dietary intake and detect associated biomarkers of food consumption becomes essential.

#### *Quizzing microbiota-diet cross-talks *via* metabolomics*

In contrast to earlier conceptualizations of personalized nutrition, which predominantly centered around genotyping, recent advancements have integrated microbiome analysis into innovative approaches to improve the efficacy of dietary and lifestyle suggestions. The core principle is to tailor dietary interventions by maximizing the diversity and abundance of intestinal microbiota [191]. Considerable interest surrounds the potential impact of diet on the composition of intestinal microbiota and the subsequent effects on the metabolome of microorganisms. The gut microbiota have the ability to metabolize the nutrition as a substrate, resulting in the production of small molecules that facilitate interactions between the microbiome and the host [192–194]. For example, some studies suggest that a considerable percentage of SCFAs produced by microbiota are absorbed by the host organism [195] and contribute to the sustenance of the host's nutritional requirements [196]. In this context, a few years ago, the objective of the FRUVEDomics Study was to identify dietary risk factors through the utilization of metabolomics and microbiome analyses. The results of the FRUVEDomics Study revealed a significant correlation between metabolic syndrome and a heightened abundance of *Firmicutes* in comparison with *Bacteroidetes* [197]. Numerous additional studies have underscored the significance of examining the intestinal microbiota in order to achieve precision nutrition [198, 199]. Furthermore, the results of this study demonstrate a positive association between increased levels of trimethylamine in fasting plasma, which is generated by the gut flora, and a heightened vulnerability to atherosclerosis. Furthermore, their findings led to the formulation of precise dietary guidelines, one of which advised individuals with a gastrointestinal microbial ecosystem capable of converting red meat-derived nutrients into compounds that promote atherosclerosis to reduce their red meat intake [200]. Additional general recommendations, such as the use of artificial sweeteners, may be subject to scrutiny. Suez and coworkers [201] have provided evidence that individuals with susceptible intestinal flora may develop glucose intolerance as a consequence of excessive sweetener consumption. However, the results reported in their research seem to be controversial due to the considerable quantity of sweetener used [202, 203].

Prebiotics represent an additional strategy for capitalizing on the potential of the microbiota-host interaction via dietary means (*i.e.*, via metabolites). Prebiotics, which are substrates that microbes inhabiting the host organism specifically metabolize, lead to advantageous health consequences for the host. The modification of the microbiome is an additional viable strategy to implement [204]. Multiple studies have suggested that prebiotics may have therapeutic applications [205–208]. The correlation between a high-fiber diet and an increased Prevotella/Bacteroides ratio, which results in improved glucose metabolism, is evident [209]. However, the domain of food science comprises an extensive array of biochemical variables, many of which are often impacted by the microbiota [210]. Consequently, by precisely modifying the microbiome composition of a given diet, it becomes feasible to tailor dietary interventions [211]. Zeevi and coworkers [212] utilized an advanced methodology in which they incorporated microbiome, clinical, and nutritional data to develop predictive models that could deliver individualized dietary suggestions for enhancing glycemic control. Shoaie et al. examined the relationship between nutrition, intestinal microbiota, and host metabolism by employing genome-scale metabolic modeling. With precision, the researchers predicted the microbiome-metabolic responses that would occur in obese individuals in response to a food intervention. After that, they examined metabolomics data from the feces and blood to validate their hypotheses [213]. Given the dynamic character of the microbiota, interventions that specifically target the microbiome have considerable potential for customizing dietary strategies to suit the needs of individual patients. Nevertheless, the efficacy of interventions that specifically target the microbiota may be hindered by the individual's previous dietary habits [214] and the initial makeup of their microbial communities [215], due to the adaptability of the microbiota. Hence, it is imperative to establish an accurate and comprehensive characterization of the diet-responsive microbiota in order to enhance the efficacy of dietary interventions.

Notwithstanding the challenges that impede the effective implementation of microbiota-related nutrition interventions in clinical settings, recent progressions in computational and analytical methodologies offer the potential for surmounting these constraints. The integration of genomics with additional omics fields, including proteomics and metabolomics, enables the achievement of more accurate and comprehensive functional profiling [212, 216]. It is imperative to undertake extensive and conclusive investigations to establish dependable and comprehensive results. In addition, it is crucial to carry out controlled investigations to precisely characterize environmental components distinct from nutrition, given that these elements might substantially impact the gut microbiota ecosystem model. These research-based standards will enable the investigation of potential prospects for the development of personalized nutritional strategies in the era of precision nutrition.

### Microbiomics versus genotyping and nutrigenomics

Nutrigenomics and microbiomics are two disciplines that emerged several years ago and have experienced significant independent growth, resulting in a substantial body of published literature and scientific research. The objective of their study is to elucidate the etiology of various diseases through the utilization of diverse methodologies and approaches. Nutrigenomics ascribes the genetic variations of Single Nucleotide Polymorphisms (SNPs) as the underlying cause of diseases. Conversely, the Microbiomics field attributes the origins of these diseases to the changes in the Microbiota that are associated with human pathophysiology.

Nutrigenomics encompasses the methodologies employed to investigate the interactions between dietary components and genes, as well as their resulting products, to modify the phenotype. Conversely, it also explores how genes and their products metabolize these constituents into nutrients and bioactive compounds. The overarching objective of nutrigenomics is to enhance health outcomes by tailoring dietary interventions to individual needs. Simultaneously, it is imperative to offer robust methodologies for comprehending the intricate interplay between nutritional molecules, genetic polymorphisms, and the entirety of the biological system [217]. The International Society of Nutrigenetics/Nutrigenomics proposes that the future of personalized nutrition should encompass three distinct levels. The first level involves conventional nutrition, based on general guidelines tailored for specific population groups, considering age, gender, and social determinants. Moving beyond this, the second level introduces individualized nutrition, which incorporates phenotypic information to assess the current nutritional status of individuals. Finally, the third level of personalized nutrition is genotype-directed nutrition, which considers rare or common gene variations when designing dietary recommendations [218].

Since different types of mutations might have diverse effects, differences in the SNPs are used to assess their effects at the gene expression level. Forecasting the consequences of a mutation on the phenotypic manifestation is a multifaceted undertaking that necessitates carefully considering all pertinent factors and their interrelationships. The interplay among genes, diet, and disease was initially observed in the 1930s when phenylketonuria, a representative rare Mendelian disorder caused by mutations in *phenylalanine hydroxylase* (*PAH*) and a deficiency in phenylalanine metabolism, was identified [219]. Researchers have made significant progress in GWASs and targeted investigations involving candidate gene panels in the last twenty years. These advancements have facilitated the expedited identification of genetic variants involved in gene-diet interactions and their potential links to various diseases. This newly acquired capability has facilitated the emergence of the field of nutrigenetics [220]. Numerous studies and trials have elucidated nutrigenetic variations linked to prevalent ailments, including colorectal cancer, obesity, T2DM, and CVD, among others [221–224].

Numerous studies conducted across diverse populations have examined the impact of multiple SNPs on weight loss, weight regain, and metabolic enhancements pertaining to serum lipid levels and insulin resistance. These investigations encompass the examination of polymorphisms located in or near genes involved in regulating food intake, lipid and lipoprotein metabolism, insulin signaling, glucose homeostasis, inflammatory response, amino acid metabolism, and circadian cycle have contributed to the exploration of these genetic variations [224–239].

An example can be found in the impact of riboflavin consumption on individuals with cardiovascular disease who possess the homozygous state for the prevalent *677C → T* variant in the *methylenetetrahydrofolate reductase (MTHRF)* gene. Following a 16-week intervention period, during which participants were administered a daily dosage of 1.6 mg riboflavin or a placebo, it was observed that riboflavin consumption led to a decrease in average blood pressure levels among individuals who possessed a homozygous genotype for the specific polymorphism under investigation [240]. Another investigation examined the impact of eicosapentaenoic acid and docosahexaenoic acid supplements on cardiometabolic factors influenced by the apolipoprotein E (*APOE*) genotype. The study revealed notable interactions between sex and genotype treatment, particularly in reducing blood triacylglycerol levels. The most pronounced effects were observed in men possessing a specific *APOE* genotype [241].

Furthermore, nutrigenetic tests have incorporated SNPs to assess their influence on modifying dietary behaviors. An illustration of the efficacy of gene-based personalized nutrition, explicitly targeting the *APOE* gene, was demonstrated to surpass conventional dietary guidance in reducing saturated fat consumption [242]. Livingstone et al. [243] reported that participants who received gene-based personalized nutrition, targeting specific variants in five nutrient-responsive genes, exhibited higher scores on the Mediterranean diet than those who received dietary advice based solely on their current diet and phenotype. Additionally, a study conducted by Nielsen and El-Sohemy [244] found that the revelation of genetic information about the *angiotensin I converting enzyme* (*ACE*) genotype, specifically in the context of personalized nutrition, led to more significant alterations in sodium consumption when compared to dietary recommendations based on the general population. Similarly, individuals who were provided with information regarding their genetic makeup related to fatty acid desaturase 1 (FADS1) exhibited a higher level of awareness regarding the significance of omega-3 fatty acids in maintaining good health. Additionally, these individuals reported encountering fewer obstacles in incorporating omega-3 fatty acids into their diet than those who did not receive personalized genetic information [245]. The study by Nielsen and El-Sohemy [246] established a connection between the comprehension, consciousness, and efficacy of genetic-based dietary recommendations compared to generic dietary guidance. Collectively, these findings prompt an important inquiry regarding the potential customization of dietary guidelines according to genetic variations and the extent to which personalized nutrition may differ from conventional recommendations in terms of its impact.

Currently, there is a growing trend among various companies to provide direct-to-consumer genetic-based nutritional testing along with corresponding guidance [247]. The exponential expansion of this sector serves as evidence that a substantial portion of consumers possess a strong desire for the perceived advantages associated with "gene-based diets". These companies provide clients with services related to genotyping and/or secondary data analysis. Subsequently, the obtained outcomes are processed to propose individualized nutritional modification strategies. Nevertheless, the emergence of this novel discipline has sparked controversy, as evidenced by the critical assessments of nutrigenetic testing companies [248–250]. However, it has been argued by Castle et al. [251] that certain criticisms lack factual basis and can potentially harm private interests. Hence, the persistent task lies in enhancing and elucidating the efficacy of genetic testing methodologies by utilizing robust scientific evidence.

Nevertheless, it is important to acknowledge that personalized nutrition is currently in its nascent phase and, in many instances, requires further scientific investigation before widespread implementation can occur. This is particularly crucial due to the intricate nature of genetic modifications, their corresponding impacts, and the limited understanding of the specific dietary factors that may trigger adverse gene-diet interactions.

Although the prevailing notion in molecular biology is that the flow of genetic information from DNA to RNA to protein is a linear and uncomplicated process, it is important to acknowledge that various modifications can occur during this progression. These modifications can potentially disrupt gene expression and subsequently impact the functional consequences of genetic variations. Although an extensive examination of gene expression or epigenetics is outside the purview of this review, it is crucial to note that individual variants may not exhibit equal expression across all individuals. The process of identifying genotypes is relatively straightforward; however, comprehending the intricate molecular and metabolic network of events influenced by a specific genetic variation is considerably more intricate. Certain SNPs possess established functions or connections with diseases or other phenotypic traits, encompassing the metabolism of dietary components and nutritional deficiencies. However, it should be noted that these variants are not commonly observed and are considered to be atypical occurrences. Furthermore, it should be noted that in instances where a clinical correlation has been established, it is important to consider that these associations may not necessarily apply to diverse racial and ethnic populations, as numerous traits exhibit significant influences from both developmental and environmental factors while displaying relatively limited heritability.

Despite extensive research on the interaction between genes and diet, the Academy of Nutrition and Dietetics's recent conclusion indicates that a limited number of randomized controlled trials are available to guide the incorporation of genetic variation into the Nutrition Care Process [252]. The limited applicability of genetic information for dietary advice stems primarily from the fact that genetic evidence is obtained through epidemiological association studies and cannot be directly extrapolated to clinical settings. An additional constraint exists in the absence of comprehensive dietary intervention studies considering an individual's genetic background. There is a need for the establishment and consensus on protocols within the scientific community to effectively identify gene variants that are both clinically and practically significant among the numerous loci that have been linked to obesity or CVD. These recommendations could subsequently be integrated into effective policy and practice guidelines for personalized nutrition. Based on a retrospective analysis of the preceding two decades, it becomes apparent that forthcoming personalized nutrition concepts necessitate enhanced input and output variables. Collecting empirical data on food consumption and various aspects of human behavior, such as physical and social activities, has become increasingly feasible due to the unprecedented capabilities of the "digital environment." In addition, there is a requirement for more extensive phenotyping and enhanced algorithms utilizing artificial intelligence to forecast the impact of an individual's dietary choices on metabolic response and risk mitigation. The enhancement of research on Personalized Nutrition could be achieved through the improved integration of data sciences and the promotion of multidisciplinary collaboration.

## Conclusions and future perspectives

The concept of personalized nutrition is rooted in the understanding that individuals possess distinct physiological and genetic characteristics that impact their bodily responses to various dietary components and nutrients. The field of nutrigenetics has experienced significant advancements, revealing that genetic variants can affect the levels of macronutrients and micronutrients and an individual's response to dietary intake. These variations hold significant value in facilitating the development of personalized dietary interventions, thereby enabling the effective translation of generic dietary guidelines into genotype-directed nutrition. Nevertheless, various obstacles could impede the extensive implementation of personalized nutrition [253]. To begin with, it is important to note that nutrition-related diseases, such as CVD and T2DM, exhibit a polygenic nature. Consequently, these diseases are influenced by the collective impact of numerous genes, each exerting modest to moderate effects. Hence, using polygenic risk scores encompassing a multitude of genetic variants may yield enhanced predictive capabilities in determining the efficacy of personalized nutritional interventions [254]. Furthermore, the manifestation of diseases is attributed to the intricate interplay between genetic predisposition and external environmental influences. For instance, individuals who possess a genetic predisposition to obesity may exhibit heightened probabilities of weight gain in comparison with the broader population when they consume equivalent quantities of sugar-sweetened beverages [229]. In this context, the utilization of sophisticated "omics" technologies, such as epigenomics, transcriptomics, proteomics, metabolomics, and microbiome analysis, in deep phenotyping holds the potential for elucidating gene-environment interactions and elucidating the unresolved hereditary factors [255, 256]. In addition, it is worth noting that "omics" technologies yield substantial volumes of data, necessitating the utilization of sophisticated analytical techniques like machine learning [257, 258]. These techniques have been employed across various facets of personalized nutrition, encompassing blood glucose monitoring [259], body weight management [260], disease risk evaluation [261], and nutritional administration [262]. In addition, the successful execution of personalized nutrition necessitates the precise involvement of healthcare practitioners and individuals' adherence, necessitating innovative digital tools or tracking devices to connect all parties [263].

In summary, research focused on genotype-based nutritional studies has underscored the significant influence of SNPs in governing the levels of macronutrients and micronutrients, which are essential for maintaining overall well-being. While additional research is needed to integrate personalized nutrition into healthcare research and practice effectively, existing evidence suggests the importance of incorporating more genetic variants into personalized nutritional interventions [264, 265]. This approach is crucial for enhancing nutrient levels, promoting better health outcomes, and alleviating the strain of nutritional disorders on healthcare services.

## Data Availability

Not applicable.
